# Serum chloride levels in critical illness—the hidden story

**DOI:** 10.1186/s40635-018-0174-5

**Published:** 2018-04-13

**Authors:** Carmen Andrea Pfortmueller, Dominik Uehlinger, Stephan von Haehling, Joerg Christian Schefold

**Affiliations:** 1 Department of Intensive Care, Inselspital, Bern University Hospital, University of Bern, Freiburgstr. 10, 3010 Bern, Switzerland; 2 Department of Nephrology, Inselspital, Bern University Hospital, University of Bern, Freiburgstr. 10, 3010 Bern, Switzerland; 3 Department of Cardiology and Pneumology, Innovative Clinical Trials Group, University of Göttingen, Robert-Koch-Str. 10, 37099 Göttingen, Germany

**Keywords:** Renal function, Intensive care, Electrolytes, Mortality, Acid-base disorder

## Abstract

Chloride is the principal anion of the extracellular fluid and vital for both serum electroneutrality and acid-base homeostasis. The aim of this review is to investigate the relevance of dyschloremia in the critically ill.

An extensive literature research was conducted on www.pubmed.org. In addition, the references of included articles were searched for further possible investigation regarding chloride.

Articles investigating the relevance of dyschloremia in the critically ill were included.

All articles were screened in regard to dyschloremia in the critically ill.

Chloride is essential for blood pressure control, decarboxylation/gas transport, renal function, and gastrointestinal homeostasis. “Dyschloremia,” i.e., serum chloride levels not within the limits of normal, may commonly be observed on ICUs and appear mainly induced by iatrogenic measures (i.e., infusion of chloride-rich fluids). Hypo- and hyperchloremia appear linked to increased mortality in defined ICU populations, but evidence is sparse. Data show that hyperchloremia may not only be linked to hyperchloremic metabolic acidosis, but also to increased hemodynamic instability and vasopressor need (e.g., in patients after major surgery). Nevertheless, it is currently unknown whether such effects would be directly or indirectly mediated. Moreover, recent evidence points to an increased incidence of acute kidney injury and need for renal replacement therapy in patients with advanced hyperchloremia.

Current knowledge on chloride is largely limited by heterogeneous trial design and mostly abundant data on specific fluid replacement strategies. The aim of this review is to summarize key consequences of chloride in critical illness and to discuss implications for daily clinical practice and future research.

## Review

The chloride ion (Cl^−^, molar mass 35.45 g/mol) is the principal extracellular anion in humans [1–3]. Intra- and extracellular chloride concentrations range from 2 to 5 mmol/L (skeletal muscles) to about 90 mmol/L (erythrocytes), and 97–107 mmol/L (plasma). Chloride is vital for maintenance of serum electroneutrality, acid-base balance, fluid homeostasis, osmotic pressure, hydrochloric acid (HCl) production in the gastrointestinal tract, renal function, and for electrical activity in general, e.g., in muscular activity [1, 2].

Hyperchloremia has a high prevalence in critically ill patients with data showing that it may be observed in about 25–45% of ICU patients; however, this seems not acknowledged by previous research or textbooks. Data from a recent prospective observational investigation demonstrate that temporary hyperchloremia may even occur in 75% of ICU patients during the first 24 h of ICU stay [4]. However, despite a rather high prevalence in critically ill patients, few outcome-related data regarding systemic chloride levels exist. The available data indicate that in general, increased disease severity is associated with abnormal chloride levels (reviewed in [1]). This review aims to provide an overview on chloride physiology and to reflect outcome-relevant effects of chloride in critically ill patients.

### Physiological functions of chloride—a quick overview

**Fig. 1:**
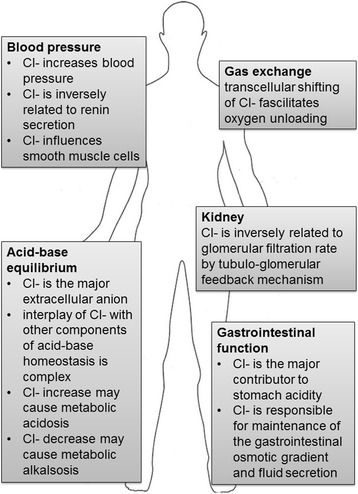
The principal physiological functions of chloride in the human body

In humans, dietary salt intake is the primary Cl^−^ source (about 6–12 g, respectively 100–200 mmol Cl^−^) [1, 5]. Cl^−^ is vital for several key physiological functions discussed below (Fig. 1).

#### Acid-base equilibrium

Cl^−^ is the major extracellular strong ion and is key to maintenance of acid-base homeostasis [1, 6, 7]. Cl^−^ levels are inversely related to bicarbonate [1], which acts as the major acid-base buffer in humans [1]. Cl^−^ was identified as the primary factor influencing the occurrence of metabolic alkalosis and non-anion gap metabolic acidosis in critical illness [6].

**Fig. 2:**
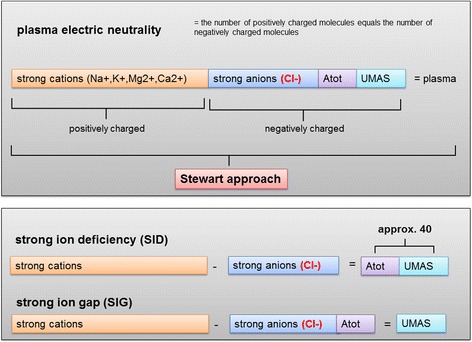
Acid-base physiology according to the Stewart’s approach

**Fig. 3:**
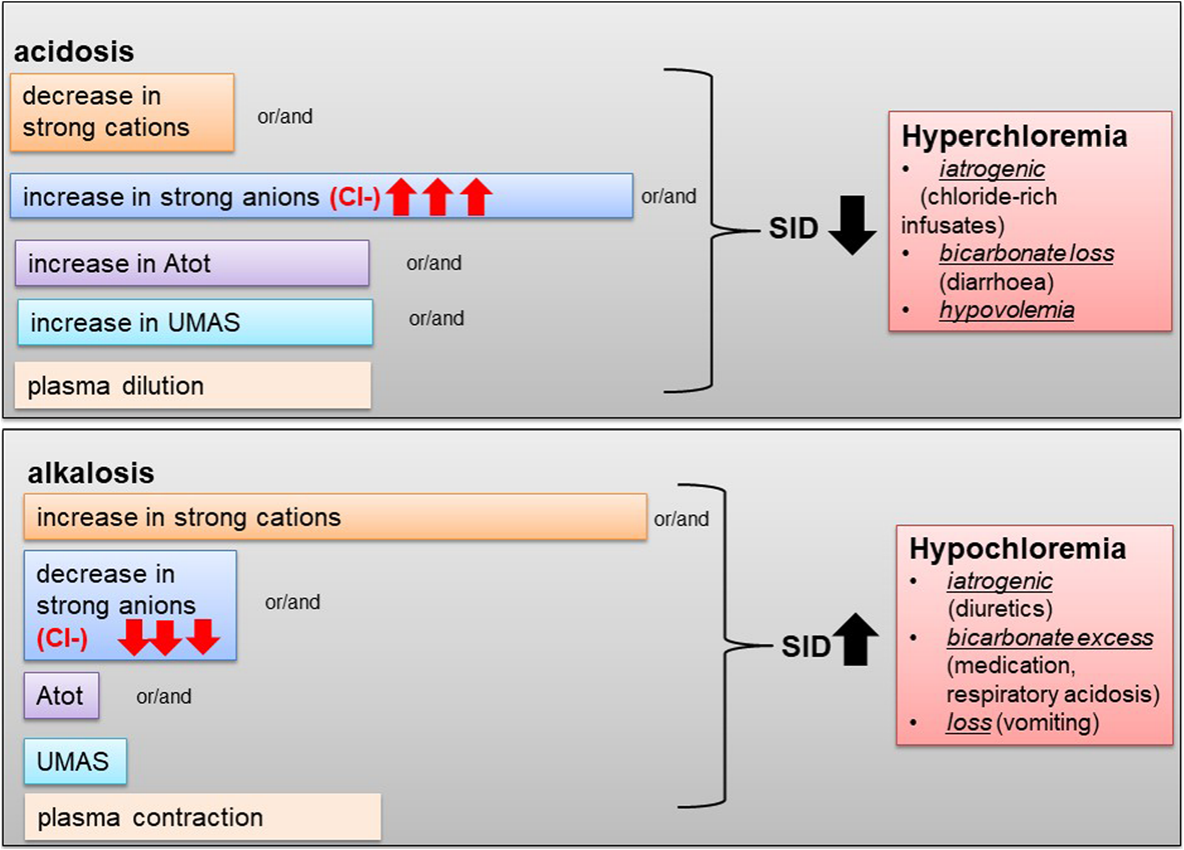
Influence of chloride on acid-base homeostasis

The influence of Cl^−^ acid base homeostasis can be explained by the “Stewart approach” (Fig. 2), where the potential proton concentration of a given solute is determined by changes in any of three independent variables: (1) difference in so-called strong ions (SID) where Cl^−^ plays a major role, (2) carbon dioxide partial pressure, and (3) non-volatile weak acid concentration [7, 8]. In addition, Cl^−^ levels are significantly influenced by compensatory factors, urine electrolyte, and bicarbonate concentrations and water homeostasis. The influence of Cl^−^ on acid-base homeostasis is provided in Fig. 3.

#### Influence of chloride on renal function and blood pressure

Cl^−^ undergoes free glomerular filtration with 99% being reabsorbed and about 180 mmol of Cl^−^ excreted per day [1, 9]. Cl^−^ reabsorption occurs in the proximal renal tubule (~ 60%) and partly in intercalated cells of the distal nephron. In the ascending part of Henle’s loop, another 25% of Cl^−^ is reabsorbed [1, 9–11]. In euvolemia, Cl^−^ levels regulate active sodium-potassium and Cl^−^ reabsorption [1] by tubular-glomerular feedback with Cl^−^ sensing in the macula densa. This feedback may induce renal afferent vasoconstriction and reduced glomerular filtration [11–14]. Moreover, Cl^−^ levels affect renin secretion and Cl^−^ concentrations at the macula densa and are inversely related to renin-angiotensin-aldosterone system (RAAS) activation [1, 12]. Several preclinical studies showed that Cl^−^ depletion induced stimulation of renin secretion resulting in increased systemic blood pressure [15]. In addition, Cl^−^ concentrations may exert direct effects on smooth muscle cells resulting in vasoconstriction [5].

#### Gastrointestinal function of chloride

Cl^−^ has two distinct functions in the gastrointestinal (GI) tract: first, it is secreted in form of HCL and is required for protein digestion, microorganism homeostasis, and absorption of nutrients (e.g., calcium, zinc, iron, vitamins, folic acid) [1]. Second, it is responsible for maintenance of the GI osmotic gradient and fluid secretion [1]. The role of Cl^−^ in splanchnic perfusion is discussed controversially with few animal data indicating that increased Cl^−^ levels lead to impaired gastric-pyloric motility, nausea, and vomiting [16].

#### Effects of chloride on oxygen transport and gas exchange

Intracellular Cl^−^ are lower than extracellular Cl^−^ levels [1]. The extracellular vs. intracellular Cl^−^ distribution mainly depends on cell membrane potentials that are established by transmembrane electrolyte transport [1]. Erythrocytes have low membrane potentials allowing for almost free transmembrane Cl^−^ passage [1] and anion exchange mainly occurs via an Cl^−^/HCO_3_^−^ antiport. The underlying physiological mechanism (Cl^−^/HCO_3_^−^exchange) is referred to as “Hamburger shift” and seems key to understanding carbon dioxide (CO_2_) transport. In fact, when blood passes through the venous system with high CO_2_ pressures, a chloride efflux and concurrent bicarbonate influx (derived from CO_2_; CO_2_ uptake) occurs which is diffused while respective blood is arterialized in the pulmonary system [17, 18]. This Cl^−^ shift plays a role in oxygen (O_2_) unloading also [17, 18]. Nevertheless, the clinical significance of this effect needs confirmation in subsequent investigations.

### Clinical conditions associated with “dyschloremia” on the ICU

#### Definition of “dyschloremia”

Hypochoremia is usually defined as serum chloride levels below 96–101 mmol/l, while hyperchloremia normally is defined as serum chloride levels higher than 106–111 mmol/l [19–21]. The definition varies depending on the local laboratory. Chloride levels do closely interact with the body’s water contact and are highly susceptible to either plasma contraction or dilution (also see below).

#### Hypochloremia

Hypochloremia in critically ill patients can be caused by active Cl^−^ loss, e.g., through the GI tract (e.g., vomiting, diarrhea), via inadequate renal Cl^−^ reabsorption or via dilution following infusion of hypotonic fluids [1, 9]. Additionally, Cl^−^ can be lost via the kidneys in cases of increased bicarbonate reabsorption in either chronic respiratory acidosis or hyper-aldosteronism. High-volume bicarbonate infusion may result in Cl^−^ being exchanged for bicarbonate in order to maintain electroneutrality [1]. Key to understanding of hypochloremia thus is assessment of potential iatrogenic effects and/or related use of diuretics [3]. Especially, the use of furosemide is clearly associated with the occurrence of metabolic alkalosis [22]. Plasma contraction further aggravates hypochloremic metabolic alkalosis especially in patients who lose high quantities of chloride-rich fluids (e.g., vomiting) [23].

#### Hyperchloremia

Hyperchloremia in critically ill patients is mainly due to (1) loss of bicarbonate through the GI or renal tract, (2) as a consequence of “dilution” due to volume loading with fluids with a low bicarbonate concentration, or (3) by excess infusion of Cl^−^-rich fluids [1]. On the ICU, diarrhea may be the most often reason for bicarbonate loss [1]. Bicarbonate may also be lost through the renal system in renal tubular acidosis (RTA), especially in proximal RTA type II [1, 24].

Further, plasma “dilution” may also decrease bicarbonate levels [25, 26]. This typically results in increased Cl^−^ levels and “dilutional acidosis”—which was also observed after infusion of large quantities of chloride-rich fluids [12, 25, 26].

Hyperchloremia in critical illness most often results from iatrogenic chloride overload (e.g., 0.9% NaCl infusion with 154 mmol/l CL^−^) [1, 12, 27–30]. Normal saline has a theoretical SID of zero [1] and thus results in development of hyperchloremic metabolic acidosis [1, 12]. Despite growing evidence, 0.9% NaCl is still one of the most widely used crystalloids [12, 30–36]; however, its use is widely debated [37–43]. Importantly, the ICU physician should note that albumin-based replacement fluids may contain rather large quantities of chloride also [44, 45]. Furthermore cases of hyperchloremia on the ICU may result from infusion of HCl, acetazolamide, and/or triamterene therapy, or specific cortisone derivates resulting in NaCl retention [1].

### Impact on clinical outcomes of critically ill patients

#### Hyperchloremic metabolic acidosis

Hyperchloremic metabolic acidosis results from infusion of considerable quantities of chloride-rich fluids in critically ill patients [27–30, 33, 35, 36, 46–48]. It’s development is dose-dependent and independent of infusion speed [13]. Importantly, hyperchloremic metabolic acidosis may not only affect ICU patients with acute kidney injury (AKI) [28], data also show that a total volume of 2000 ml of chloride-rich infusate may induce hyperchloremic metabolic acidosis in healthy volunteers [13]. Hyperchloremic metabolic acidosis may induce vasodilatation [49–51], altered neurotransmitter function [52, 53], decreased cardiac reactivity [52, 53], and other changes in cellular function [54], as well as decreased endogenous catecholamine release [55].

Current literature clearly indicates that chloride-rich infusates are associated with the temporary occurrence of hyperchloremic metabolic acidosis. However, the significance of the latter and its influence on clinical endpoints such as the occurrence of kidney failure or mortality is not jet clarified.

#### Renal function

Effects of hyperchloremia on renal function were first investigated over 30 years ago [11, 14]. There is some animal [11, 14, 47] and human data (13) that suggest that renal blood flow and renal cortical perfusion is diminished under chloride infusion. However, a recently published trial [56] does not confirm these findings.

Clinical studies, like animal experiments, showed mixed results regarding patient-centered clinical outcomes (e.g., need for renal replacement therapy (RRT)) in ICU patients [4, 33, 35, 37, 46, 57–62] (see, Table 1). Whereas some clinical trials did not identify changes in serum creatinine or acute kidney injury (AKI) rates in mixed ICU cohorts, cardiac surgery, or sepsis [37, 59, 62], other reports demonstrate increased AKI incidence and need for renal replacement therapy (RRT) [4, 46, 57]. However, the sensitivity analysis of one of these trials showed that the incidence of AKI and need for renal replacement therapy were also influenced by other unidentified confounders [57], so the issue is far from being concluded. This is also confirmed by another recently published retrospective analysis comparing hypertonic (3%) to normal saline in patients undergoing emergent laparotomy that showed no difference in respect to renal outcomes between the groups, even though the chloride levels were significantly higher in the hypertonic saline group [63].

**Table 1:** Overview on studies investigating the impact of hyperchloremia on renal function

Author (year)	Design	Study population	Total study population	Study intervention	Increased incidence of acute kidney injury	Increased need for renal replacement therapy	R
Krajewski ML et al. (2015)	Meta-analysis	Perioperative patients	6253	0.9% saline versus other crystalloid infusates	X	Not investigated	[33]
Shaw B et al. (2012)	Retrospective cohort study	Patients after open abdominal surgery	31,920	0.9% saline versus other crystalloid infusates	X	Not investigated	[35]
Young et al. (2015)	RCT	General ICU population	2278	0.9% saline versus acetate-buffered solute	No difference between the groups	No difference between the groups	[37]
Yunos et al. (2012)	Open label, sequential period study (6 month)	General ICU population	760	Chloride-rich versus chloride-depleted infusion solutes	X	X	[46]
Yunos et al. (2015)^a^	Open label, sequential period study (1 year)	General ICU population	2994	Chloride-rich versus chloride-depleted infusion solutes	X	X	[57]
Guirgis FW et al. (2015)	Retrospective cohort study	Patients with sepsis/septic shock	95	Chloride-rich versus chloride-depleted infusion solutes	No difference between the groups	No difference between the groups	[59]
Shao M et al. (2016)	Retrospective cohort study	General ICU population	6025	–	X	Not investigated	[60]
Mattinen E et al. (2016)	Subgroup analysis of RCT	General ICU population	445	–	X	Not investigated	[4]
McCluskey SA (2013)	Retrospective cohort study	Non-cardiac surgery	22,851	–	X	Not investigated	[58]
Suetrong B et al. (2016)	Retrospective cohort study	Patients with sepsis/septic shock	240	–	X	Not investigated	[61]
Zhang Z et al. (2013)	Retrospective cohort study	General ICU population	1221	–	X	X	[64]
McIlroy D et al. (2017	Open label, sequential period study	Perioperative patients	1136	chloride-rich versus chloride-depleted infusion solutes	No difference between the groups	No difference between the groups	[62]
Sadan O et al. (2017)	Retrospective cohort study	Patients with subarachnoid hemorrhage	1267	–	X	Not investigated	[65]
Loftus TJ et al. (2017	Retrospective cohort study	Patients undergoing emergent laparatomy	189	0.9% saline versus 3% saline	No difference between the groups	No difference between the groups	[63]

•Sensitivity analysis showed that multiple unknown confounders may have influenced the incidence of AKI and need for RRT in this studySensitivity analysis showed that ^a^ multiple unknown confounders may have influenced the incidence of AKI and need for RRT in this study

X = finding was found

In addition, unfortunately, methodology, terminology, amount of total volume applied, and triggers for RRT differed considerably between trials. Overall, it appears that trials with lower total amount of Cl^−^ infusion (i.e., 1–2 L/24 h) found unaffected AKI rates [37, 59], whereas trials with higher infusion rates showed an increased AKI incidence and RRT need [4, 35, 46, 57] suggesting a dose-dependent effect. Despite the enormous heterogeneity in the available literature which makes it almost incomparable, a recently published meta-analysis [33] included randomized and non-randomized trials concluded that use of chloride-rich fluids is associated with a higher AKI risk.

Interestingly, increased serum Cl^−^ levels alone, independent of i.v. fluid, were associated with a higher AKI risk in several studies [4, 64, 65]. As to chloride levels, studies show that only minimal and/or maximum Cl^−^ levels during ICU stay [61] but not ICU admission levels [64] were associated with an increased AKI incidence. An increase of Cl^−^ serum levels by 10 mmol/l resulted in OR 7.39 for AKI development in one study [65]. However, the number of study focusing on chloride levels independent of i.v. fluids is still a few.

In conclusion, despite the number of studies focusing on the development of AKI and need for RRT in patients receiving chloride-rich infusates, the debate is far from being decided due to the large heterogeneity in the available literature.

#### Cardiovascular function

Chloride-rich infusions may lead to hemodynamic instability [27, 28, 31, 47]. Hemodynamic effects of chloride-rich fluids were first described by Kellum and coworkers in a rodent sepsis model [47]. In this model, hyperchloremia and associated metabolic acidosis induced decreased arterial pressures [47]. This effect was confirmed in additional studies showing decreased mean arterial blood pressures and cardiac index in rats with abdominal sepsis [31]. In critically ill humans, patients receiving chloride-rich infusions had a volume-dependent increased vasopressor need [27]. A randomized controlled double-blind study by members of our group focusing on normal saline when compared to an acetate-buffered infusion solution in patients undergoing major abdominal surgery even shows, that the effect is not only volume-dependent, but also time-dependent [66]. The mechanisms behind this effect remain somewhat elusive. Further trials comparing other infusion solutes in respect to cardiovascular stability are certainly needed before drawing definitive conclusions.

Hypochloremia seems of particular importance in heart failure patients where low levels of serum chloride indicate advanced disease, and are associated with decreased left ventricular ejection fraction [38, 67–69], increased cardiac function markers (e.g., NT-pro-BNP) [38, 68], and circulating catecholamine levels [70]. In fact, hypochloremia was identified as an independent predictor for adverse outcome in heart failure patients and was recently recognized to predict mortality in affected patients [3].

The influence of chloride on the cardiovascular system may be important for clinicians for several reasons: first, chloride loading may contribute to catecholamine need in critically ill patients. Second, cardiac function may be influenced by chloride levels in a “U-shaped” response curve with both hypo- and hyperchloremia being detrimental for cardiovascular stability (and function). Third, the effect of chloride-“loading” on hemodynamic stability may be dose-dependent. Fourth, in preclinical models, it was shown that simple hyperchloremia may trigger increased blood pressures. However, only concomitant hyperchloremia with metabolic acidosis results in decreased systemic pressures [47]. It thus seems likely that the occurrence of acidosis and not hyperchloremia per se is responsible for observed adverse cardiovascular effects.

#### Inflammation and coagulation

In several animal models, systemic levels of inflammatory makers were increased following chloride-rich infusions. This was observed in both experimental sepsis [12, 39, 47, 71] and trauma models [40]. In humans, this remains controversially discussed [41, 42] as effects of chloride-rich infusions on inflammatory markers may also be attributed to sodium rather than to Cl^−^. However, this requires further clarification.

Preclinically, chloride-rich infusions were associated with an increased need for blood products [35, 40, 43]. Moreover, few evidence points to the fact that hyperchloremia may influence plasmatic coagulation cascades [40, 43, 72] and/or platelet function [12]. In humans, several trials and a recent meta-analysis demonstrate increased need for blood product administration in patients receiving chloride-rich infusions [33, 35, 73]. Nevertheless, the effect of acidosis in this context remains uncertain.

#### Mortality and other patient-centered clinical outcomes

**Fig. 4:**
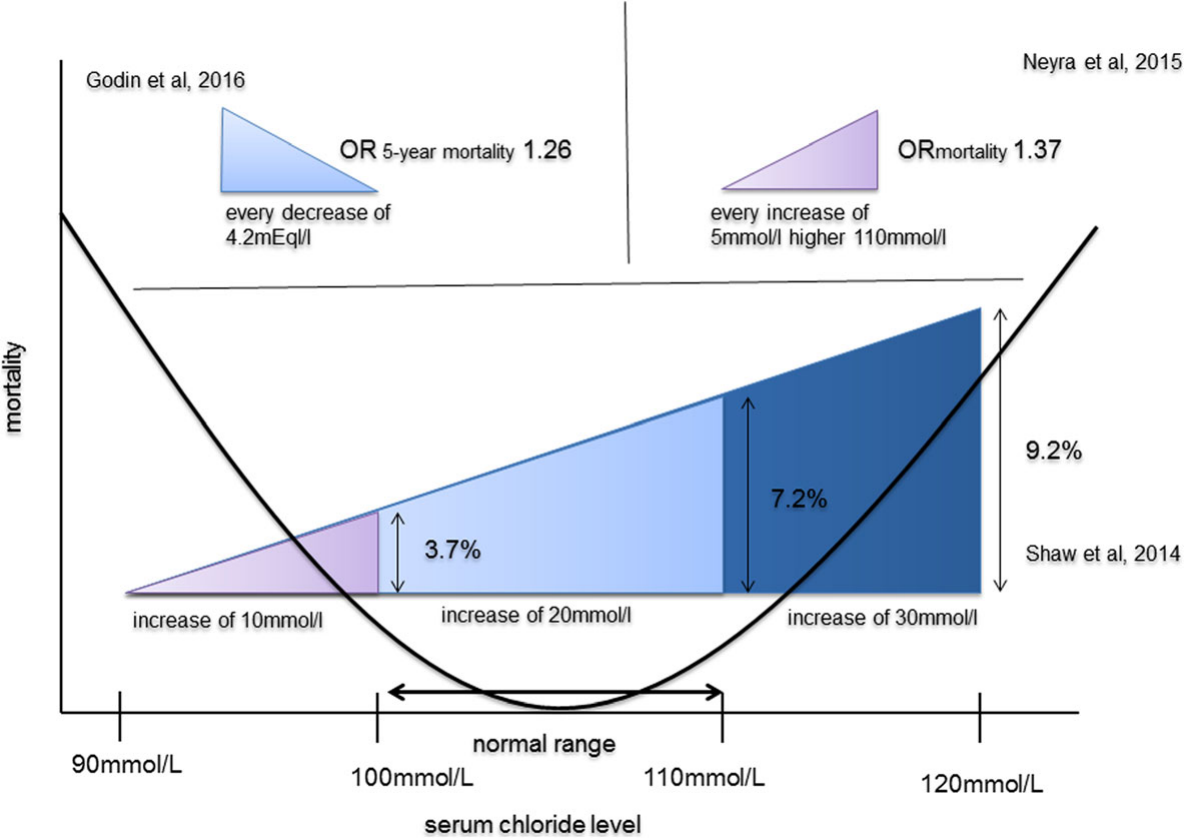
Influence of chloride levels on mortality. *All tables and figures are propriety of the authors and have not been published elsewhere

A U-shaped mortality curve was reported in respect to Cl^−^ levels and mortality [12]. This is depicted in Fig. 4. Several large-scale clinical trials in critically ill patients found increased mortality rates in patients treated with chloride-rich infusions [2, 29, 33, 35, 46, 48, 58, 74, 75]. However, this effect was not confirmed in four other large-scale multi-center trials and a recent meta-analysis [4, 33, 37, 46, 76]. Even when a very high Cl^−^ load fluid (hypertonic saline, 3%) was compared to normal Cl^−^ load fluid (0.9% saline), there was no difference in respect to mortality between these groups [76].

Hyperchloremia itself (at 72 h after ICU admission) or rise in Cl^−^ levels of > 5 mEq/l was associated with increased in-hospital mortality [2]. Interestingly, two large studies in SIRS patients [29, 48] found that mortality remained lowest in patients with only minimal serum Cl^−^ variation during total hospital stay [29]. In-hospital mortality gradually increased with each 10 mmol/l of serum Cl^−^ level increase [29]. This association was independent of total fluid volume administered, but linked to volume-adjusted chloride-load [29]. Another study in critically ill patients investigating the prognostic potency of acid-base variables to reflect in-hospital mortality identified hyperchloremia and hypoalbuminemia as the only independent factors after adjustment [77].

In conclusion, even though literature points towards increased mortality rates in patients with hyperchloremia, it remains unclear whether potential effects of Cl^−^ levels on mortality are due to direct or indirect (e.g., acidosis) effects.

Hypochloremia was also linked to mortality in several studies [38, 67, 68, 70, 75, 78] and may be of special importance in patients with heart failure. An inverse relationship of Cl^−^ levels with mortality in patients with compensated and non-compensated heart failure was shown [3, 38, 67]. Some studies showed an independent effect of hypochloremia (< 100 mmol/l) on cardiovascular, non-cardiovascular, and all-cause mortality [38, 67, 79]. All-cause mortality rates were increased even after 5 years of follow-up following initial hypochloremia [67]. Moreover, a recent editorial concludes that reduced serum Cl^−^ levels may be of higher prognostic importance than increased sodium levels in heart failure patients [3].

Unlike with hyperchloremia, hypochloremia is more closely associated with increased mortality and should certainly be considered by intensive care physicians.

Effects of chloride levels on several other patient-centered relevant clinical outcomes were investigated. Hyperchloremia was associated with increased length of mechanical ventilation [33], increased rates of post-operative infectious complications [35, 48], increased readmission rates [48], and increased ICU and hospital length of stay [48, 58, 78]. However, these outcomes need to be further evaluated before drawing any definitive conclusions.

## Conclusions

Hypo- or hyperchloremia may often be observed on ICUs, but data on relevant patient-centered clinical outcomes remain sparse. In fact, most studies investigating “dyschloremia” were heterogeneous and hyperchloremia was a result of infusion of normal saline (and thus concomitant sodium infusion). Moreover, different laboratory methods to measure Cl^−^, definitions of hypo- and/or hyperchloremia, different trial design/methodology, and cohorts under investigation add to a considerable heterogeneity of the available data.

“Dyschloremia,” however, may have a major impact on clinical outcomes in critical illness. Despite growing evidence favoring avoidance of chloride-rich infusions, e.g., 0.9% NaCl is still one of the most widely used crystalloid and some authors argue that despite development of hyperchloremic metabolic acidosis, its clinical significance remains elusive. In respect to renal function, the influence of hyperchloremia on renal function remains somewhat controversial and the available literature very heterogenic and almost incomparable. Therefore, no final conclusions on the topic of AKI incidence and need for RRT in respect to i.v. fluids with elevated chloride-content can be drawn. For cardiovascular aspects, growing evidence indicates that hyperchloremia-associated metabolic acidosis may induce hemodynamic instability. Hyperchloremia also may also have negative effects on coagulation cascades and increased mortality.

Interestingly, hypochloremia was much less studied than hyperchloremia although emerging evidence shows that low chloride levels may largely affect outcome, especially mortality in patients with heart failure.

In conclusion, “dyschloremia” significantly influences several important outcomes in the critically ill. However, much of the discussion is subject to an ongoing debate.
